# Biological and Host Range Characteristics of *Lysathia flavipes* (Coleoptera: Chrysomelidae), a Candidate Biological Control Agent of Invasive *Ludwigia* spp. (Onagraceae) in the USA

**DOI:** 10.3390/insects12050471

**Published:** 2021-05-19

**Authors:** Angelica M. Reddy, Paul D. Pratt, Brenda J. Grewell, Nathan E. Harms, Ximena Cibils-Stewart, Guillermo Cabrera Walsh, Ana Faltlhauser

**Affiliations:** 1 USDA-ARS, Invasive Species and Pollinator Health Research Unit, Western Regional Research Center, 800 Buchanan St., Albany, CA 94710, USA; Paul.Pratt@usda.gov; 2 USDA-ARS, Invasive Species and Pollinator Health Research Unit, University of California Davis, Department of Plant Sciences Mail Stop 4, One Shields Ave, Davis, CA 95616, USA; Brenda.Grewell@usda.gov; 3 US Army Engineer Research and Development Center (ERDC), Aquatic Ecology and Invasive Species Branch, 3909 Halls Ferry Rd, Vicksburg, MS 39180, USA; Nathan.E.Harms@erdc.dren.mil; 4 Instituto Nacional de Investigación Agropecuaria (INIA), Estación Experimental INIA La Estanzuela, Ruta 50 Km 11, Colonia del Sacramento, Colonia, Uruguay; xcibils@inia.org.uy; 5 Fundación para el Estudio de Especies Invasivas (FuEDEI), Simón Bolívar 1559, Hurlingham (CP1686), Buenos Aires B1686EFA, Argentina; gcabrera@fuedei.org (G.C.W.); anafaltlhauser@gmail.com (A.F.); 6 Consejo Nacional de Investigaciones Científicas y Técnicas (CONICET), Godoy Cruz 2290, Ciudad Autónoma de Buenos Aires C1425FQB, Argentina

**Keywords:** aquatic weeds, invasive species, management, host specificity, development

## Abstract

**Simple Summary:**

Exotic water primroses (*Ludwigia* spp.) are aggressive plant invaders in aquatic ecosystems worldwide. Management of exotic *Ludwigia* spp. is limited to physical and chemical control methods. Biological control, the use of insects to control exotic plants, is an alternative approach for the management of exotic *Ludwigia* spp. However, little is known regarding the natural enemies of these plants in their native range in South America. In this study, we investigated the biology and host range of a natural enemy, the flea beetle *Lysathia flavipes*, to determine its suitability as a biocontrol agent for exotic *Ludwigia* spp. in the USA. The beetle matures from egg to adult in approximately 20 days at 25 °C. Females lived approximately 86 days and laid 278–2456 eggs over their lifespans. No-choice development and oviposition tests were conducted using four exotic *Ludwigia* species and seven native USA plant species. The beetle showed little discrimination between plant species: larvae aggressively fed and completed development, and females laid eggs on most plant species regardless of origin. These results indicate that the beetle is not sufficiently host-specific for further consideration as a biological control agent of exotic *Ludwigia* spp. in the USA and further testing is not warranted.

**Abstract:**

Exotic water primroses (*Ludwigia* spp.) are aggressive invaders in aquatic ecosystems worldwide. To date, management of exotic *Ludwigia* spp. has been limited to physical and chemical control methods. Biological control provides an alternative approach for the management of invasive *Ludwigia* spp. but little is known regarding the natural enemies of these exotic plants. Herein the biology and host range of *Lysathia flavipes* (Boheman), a herbivorous beetle associated with *Ludwigia* spp. in Argentina and Uruguay, was studied to determine its suitability as a biocontrol agent for multiple closely related target weeds in the USA. The beetle matures from egg to adult in 19.9 ± 1.4 days at 25 °C; females lived 86.3 ± 35.6 days and laid 1510.6 ± 543.4 eggs over their lifespans. No-choice development and oviposition tests were conducted using four *Ludwigia* species and seven native plant species. *Lysathia flavipes* showed little discrimination between plant species: larvae aggressively fed and completed development, and the resulting females (F1 generation) oviposited viable eggs on most plant species regardless of origin. These results indicate that *L. flavipes* is not sufficiently host-specific for further consideration as a biocontrol agent of exotic *Ludwigia* spp. in the USA and further testing is not warranted.

## 1. Introduction

*Ludwigia* L. is a large monophyletic genus of wetland plant species within the Onagraceae family, currently classified into 23 sections with 88 taxa, including 83 species [1,2,3]. Most species (80%) are native to the New World, although the genus is pantropical with some (largely naturalized) representation in temperate Europe, Africa, and Eurasia [1]. Of particular interest is a group of *Ludwigia* spp. from the largely aquatic *Ludwigia* section *Jussiaea* [4], native to South America [1,3]. They have invaded both aquatic and riparian ecosystems in many regions worldwide [5,6] and are now considered among the most aggressive weeds in the world [7]. In the USA, four *Ludwigia* section *Jussiaea* taxa have naturalized in aquatic systems of the South Atlantic, Gulf, and/or Pacific coastal states [8]: *Ludwigia hexapetala* (Hook. & Arn.) Zardini, Gu & P. H. Raven; *Ludwigia peploides* (Kunth) P. H. Raven subsp. *peploides*; *Ludwigia peploides* (Kunth) P. H. Raven subsp. *montevidensis* (Spreng.) P. H. Raven; and *Ludwigia grandiflora* (Michx.) Greuter & Burdet. Rapid biomass production of these species impacts ecological processes in aquatic ecosystems, displacing desired native wildlife and vegetation [5,9,10,11,12]. Characteristic dense mats over the water’s surface also impede navigation and interfere with recreational activities, irrigation, drainage, and agricultural production [5,8].

Management of invasive *Ludwigia* spp. in the USA has to date relied on physical and chemical methods [5]. These options only provide short-term control and require repeated annual treatments, and some can be limited by regulatory restrictions in environmentally sensitive systems [8,13,14]. *Ludwigia hexapetala* and *L. peploides* also produce viable seeds with a high capacity for germination under a wide range of temperatures [15,16], resulting in persistent seedbanks that require long-term management programs [10]. Classical biological control provides a sustainable alternative to these conventional techniques as it can be used alone or as a tool in integrated management programs. Unlike other control methods, successful biological control might provide permanent, landscape-level weed suppression if exotic natural enemies are carefully selected and establish upon introduction [17].

Recently, a biological control program targeting exotic *Ludwigia* spp. was initiated by the United States Department of Agriculture’s Agricultural Research Service (USDA-ARS) at the request of water management stakeholders [18]. However, interest in biological control of exotic *Ludwigia* spp. is not new. The first foreign explorations and field host range evaluations of insect herbivores associated with *Ludwigia* spp. were conducted in the 1970s in Argentina by Cordo and DeLoach [19,20]. More recently, an expanded comprehensive survey conducted by scientists from the Fundación para el Estudio de Especies Invasivas (FuEDEI) identified 19 insect species across 6 feeding guilds that feed on *L. hexapetala* in Argentina [21]. An insect commonly observed on *Ludwigia* spp. in these surveys includes the bronze-colored, flea beetle *Lysathia flavipes* (Boheman) (Coleoptera: Chrysomelidae). Adults feed and oviposit eggs singly or in masses on leaves of their host plant [20]. All larval stages feed, develop, and pupate on leaves. Feeding by both adults and larvae can cause significant damage to host plants under high population densities [20]. Moreover, Cordo and DeLoach [20] conducted preliminary studies on the biology and host range of *L. flavipes* and concluded that the flea beetle may be suitable for introduction into the USA, but they emphasized the need for additional research as their work focused solely on adult feeding in multiple-choice tests. This, coupled with the renewed interest in *Ludwigia* biological control, led to surveys in Argentina and Uruguay during March of 2019, with a specific focus on collecting and colonizing *L. flavipes* for further study.

The primary objective of this research was to investigate the host range of *L. flavipes* in relationship to exotic and native *Ludwigia* spp. in the USA. To accomplish this goal, both no-choice and multiple-choice host range tests were conducted with an initial suite of 11 plant species that represent the target weeds (*n* = 3), as well as selecting closely related natives (*n* = 7) and one exotic species. Additionally, the life-history parameters (i.e., survival, development, and fecundity) of *L. flavipes* were collected to aid in interpreting herbivore performance across host plants. Herein we test the hypothesis that *L. flavipes* is host specific to species within the *Jussiaea* section of the genus *Ludwigia* given that there are no native representatives of the *Jussiaea* in the USA [18].

## 2. Materials and Methods

### 2.1. Origin and Rearing of Lysathia flavipes

*Lysathia flavipes* adults were collected from *L. hexapetala* plants near Punta Del Diablo (34°00′47.0″ S, 53°35′53.9″ W) and Lascano (33°44′53.9″ S, 54°07′55.4″ W) in Uruguay during March 2019, and mixed across sites. The nascent *L. flavipes* colony was exported from Uruguay under the scientific collection permit N° 9/2019 supplied by the Dirección Nacional de Medio Ambiente (DINAMA) and imported under USDA APHIS-PPQ permit # P526P-19-03070 to a USDA-ARS containment facility in Albany, California. Species identity was confirmed by the USDA-ARS Systematic Entomology Laboratory at the Smithsonian Institution, National Museum of Natural History, Washington, DC, USA. The colony was maintained on a laboratory benchtop in the Albany containment facility under ambient temperature conditions (20–25 °C). Adults were kept in 946 mL plastic containers with a piece of fine mesh cloth integrated into the lid to allow air circulation and prevent condensation. Approximately 15 adults per container fed and reproduced on a bouquet composed of three 15 cm-long excised *L. hexapetala* stems inserted into a plastic floral water tube. Stems were changed weekly. In-between feedings, water was added to the floral tubes as needed to maintain plant turgor. Periodically, older bouquets harboring eggs were retained and reared to augment colony numbers. Colonies were reared exclusively on *L. hexapetala*, originally collected from the Sacramento–San Joaquin River Delta in northern California (38°00′08.8″ N, 121°34′06.9″ W). All subsequent biology and host range studies were conducted in an environmental chamber set to a constant 25 °C (±1 °C), with a 14:10 h (L:D) photoperiod.

### 2.2. Egg Development of Lysathia flavipes

Fresh bouquets of *L. hexapetala* stems were provided to all adult colony containers. Plant material was removed after 24 h and all eggs were collected. Eggs from different clusters were mixed and spread across 15 replicate Petri dishes (90 mm diameter). Each Petri dish contained 10 eggs that were carefully placed on sterile filter paper (Whatman No. 2) moistened with water prior to sealing the dish with Parafilm^®^ to avoid desiccation. Replicated Petri dishes were arranged in a completely randomized design in a chamber and their position was rotated daily when egg hatching was monitored. Water was added as needed to keep the filter paper moist. The mean development time (days) and hatching rate (proportion) per Petri dish were calculated. Additionally, egg length (as proxy of larvae size) was measured from 30 randomly selected eggs that originated from different parental females using a dissecting microscope.

### 2.3. Preimaginal Development of Lysathia flavipes

Twenty neonate larvae (≤24 h) from the egg development study were individually transferred using a fine brush onto the young leaves of an *L. hexapetala* stem (10 cm) inserted into a floral water tube situated within an enclosed 237 mL plastic container. Fresh stems were provided weekly and water in the floral tube was replenished three times per week. Larvae were monitored daily and developmental stage (visualized by the presence of exuviae) was recorded until adult metamorphosis. On the day each molt occurred, head capsule size was measured on the greatest width of the head (genae) using a dissecting microscope equipped with an ocular micrometer. Subsequently, the number of instars, head capsule size of each instar (mm), and development time (days) of each stage were calculated. The total development time from egg to adult was calculated by adding the mean egg, larval, and pupal development times. Finally, the length of 40 randomly selected adults (20 females and 20 males) were measured from the most forward part of the head (at the frons between the eyes) to the last abdominal segment.

### 2.4. Fecundity of Lysathia flavipes

Newly molted adults from the larval development study were collected and combined in 15 replicate groups of one female and two males. Each group was enclosed in a 473 mL plastic container with a 10 cm-long *L. hexapetala* stem inserted into a floral water tube. The *L. hexapetala* stem was replaced daily and examined for eggs under a dissecting microscope. Once oviposition was confirmed, the *L. hexapetala* stem was replaced three times per week and eggs laid on the old stem were counted. This process was continued until the death of the female. If a male died, it was replaced with another male from the colony. Measures of pre-oviposition and oviposition periods, as well as longevity were recorded. Mean daily fecundity during the oviposition period and total fecundity were calculated for each female.

### 2.5. Host Range Experiments: Test Plants

A subset of the *Ludwigia* taxa and close relatives were used to provide insights into the suitability of *L. flavipes* as a biocontrol agent in the USA. The test plant list was comprised of eleven taxa from the Onagraceae: three exotic *Ludwigia* targets (*L. hexapetala*, *L. peploides* subsp. *peploides*, and *L. peploides* subsp. *montevidensis*) and seven native taxa (*Ludwigia polycarpa* Short & Peter, *Ludwigia repens* J. R. Forst., *Ludwigia palustris* (L.) Elliott, *Epilobium ciliatum* Raf. subsp. *ciliatum*, *Epilobium canum* (Greene) P. H. Raven, *Clarkia amoena* (Lehm.) A. Nelson & J. F. Macbr., and *Oenothera elata* Kunth subsp. *hookeri* (Torr. & A. Gray) W. Dietr. & W. L. Wagner). We also tested *Ludwigia decurrens* Walter, an additional congener with biogeographical similarity to the target plants that is presumed native to eastern-central USA. *Ludwigia decurrens* is non-native to California where it established approximately ten years ago as an invasive noxious weed in rice fields [22]. The native test plants (non-targets) were selected based on their range in phylogenetic relationship to the three target plants [18]. All test plants were used in both no-choice and multiple-choice host range experiments. Plants were propagated over time in a greenhouse under controlled temperature (20–32 °C), with a 14:10 h (L:D) photoperiod and ambient humidity conditions and incorporated into the host range tests as available, with *L. hexapetala* replicated in each test as the control.

### 2.6. No-Choice Development and Oviposition Tests

Four neonate larvae (≤24 h) were randomly assigned a host plant species and transferred with a fine brush onto the young leaves of a 10 cm-long stem (experimental unit) inserted into a floral water tube. Five replicate stems were individually placed in a 473 mL plastic container (4 neonates × 5 replicate stems = 20 larvae per test plant species). Larvae were transferred to fresh stems of their assigned test plant species twice per week. Water in the floral tubes was replenished three times per week during which time the larvae were observed under a dissecting microscope to record survival and developmental stage. Larval survival rate (proportion) and mean development time (*n* = 4 larvae per replicate) from 1st instar to adult was calculated for each replicate stem.

The resulting adults from the no-choice development tests were collected and grouped by emergence date. Following the colony-rearing methods described above, adults were fed and kept in a rearing container for one week to allow sexual maturation and mating. Gravid females were identified by conducting 48 h oviposition tests, then each was paired with two males and placed in a 473 mL plastic container together with a bouquet (2–3 10 mL-long stems) from the test plant the female was reared on. This process was repeated until five replicate females per test plant were evaluated. If there were not enough males from the experiments described above, adult males from the colony were used. Eggs were collected from each bouquet after seven days and counted under a dissecting microscope. Eggs from each female were then placed on moistened filter paper in individual Petri dishes sealed with Parafilm^®^. Egg viability (hatching) was monitored three times a week until all eggs hatched or became deflated (indicating mortality). Water was added to the filter paper as needed during monitoring. Subsequently, the number of eggs oviposited and egg viability (hatching rate: eggs hatched/eggs oviposited) was calculated for each replicate female.

### 2.7. Multiple-Choice Oviposition Tests

Eight adult pairs were collected from the colony and placed in a plastic container (36 L × 28 W × 24 H cm) together with 3–5 bouquets (one bouquet per test plant). Because the 11 test plant species were not available simultaneously, this study was conducted in three separate trials where a different set of plant species, including *L. hexapetala* as the control, was tested in each trial (i.e., 5, 5, and 3 plant species per trial). Five replicate bouquets per plant species were assessed in each trial (5 replicates × 11 plant species = 55 bouquets total). Each bouquet was composed of three 15 mL-long stems from a single test plant species inserted into a floral water tube as a potential source for feeding and oviposition. The side walls of the container were modified with a piece of fine mesh cloth to allow air circulation and prevent condensation within the container. Adults were collected and returned to the colony after a five-day oviposition period and eggs oviposited on each bouquet were quantified. The presence of feeding damage was noted, but not quantified.

### 2.8. Data Analyses

One-way ANOVAs were used to compare body length and longevity between female and male adults, and differences in larval survival, mean larval development time, eggs per female, and egg viability (hatching rate per female) between test plant species in no-choice tests. A Linear Mixed Model was used to test the effect of plant species on oviposition (eggs per plant) with trial as a blocking factor in the multiple-choice tests. Plant species was a fixed effect factor in all models. Post-hoc pairwise comparisons between test plants were made with Tukey’s HSD (α = 0.05). Plants species on which larvae failed to survive were omitted from the analyses. Data were tested for normality using Shapiro–Wilk tests. Egg hatch rate and larval survival data were arcsine square-root transformed, and eggs per female, eggs per plant, and adult body length data were square-root transformed to normalize results prior to analyses. All analyses were conducted using SAS Software (JMP^®^, version 13).

## 3. Results

### 3.1. Life History of Lysathia flavipes

*Lysathia flavipes* completes seven stages during development: egg, three larval instars, prepupa, pupa, and adult. Generation time (egg to adult) was 19.9 ± 1.4 d when feeding on *L. hexapetala* (range 18.2–22.2 d; *n* = 20; hereafter means are reported with ±1SD) at 25 °C.

The eggs are generally elongated oval in shape, with symmetrical round poles. The mean length was 0.7 ± 0.1 mm (range 0.58–0.82 mm; *n* = 30). The mean development time from oviposition to hatch was 4.2 ± 1.3 d (range 3–8 d; *n* = 15), and an average of 0.75 ± 0.27 (range 0.1–1; *n* = 15) of those eggs were viable.

Larvae are mobile, dark grey to black in color, and feed on all leaf stages. The average development time of the 1st, 2nd, and 3rd larval instars were 3.5 ± 0.0, 2.3 ± 0.9 (range 1.5–4.5 d), and 3.9 ± 0.6 (range 3–5 d) d, with an average head capsule size of 0.28 ± 0.02, 0.44 ± 0.04, and 0.69 ± 0.03 mm, respectively (*n* = 20 for larval development and head capsule size).

When the 3rd instar larva ceases feeding, it attaches itself to a surface (typically a leaf but occasionally the cage wall) and undergoes a prepupal period averaging 2.3 ± 0.6 d (range 1–3 d; *n* = 20). The mean pupal period was 3.9 ± 0.6 d (range 3–5 d; *n* = 20). Adult body length differed between females and males (F_1, 38_ = 1486.98, *p* < 0.0001). On average, females are nearly twice as long (8.2 ± 0.4 mm; range 7.5–9 mm; *n* = 20) as males (4.2 ± 0.2 mm; range 3.75–4.6 mm; *n* = 20).

**Figure 1:**
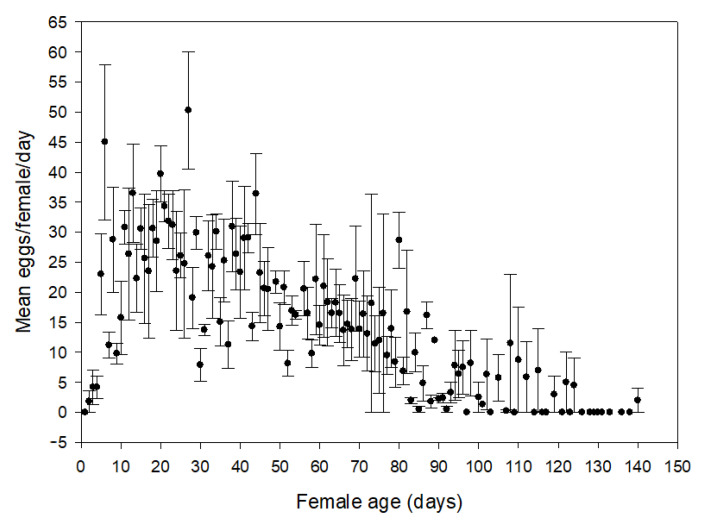
Lifetime egg production of *Lysathia flavipes* females (*n* = 15) on *Ludwigia hexapetala*.

Adults lived an average of 89.8 ± 40.8 d (*n* = 30) in the laboratory, but there was no difference between average female (86.3 ± 35.6 d; range 18.5–139.5 d; *n* = 15) and male (93.3 ± 46.4 d; range 11–161 d; *n* = 15) longevity (F_1, 28_ = 0.22, *p* = 0.65). The female undergoes a preoviposition period of 4.3 ± 1.3 d (range 3–6 d; *n* = 15). The mean number of eggs oviposited by females was 1510.6 ± 543.4 (range 278–2456; *n* = 15), with an average 19.3 ± 5.5 eggs per female per day (range 10.2–29.2 eggs per female per day; *n* = 15). Most of the eggs are laid in the first 90 days of the females’ lifetime (Figure 1).

### 3.2. No-Choice Host Range Tests

*Lysathia flavipes* larvae did not survive on two native (*E. canum* and *O. elata* subsp. *hookeri*) and one non-native (*L. decurrens*) test plant species but successfully completed development on the remaining eight species tested (Table 1). Larval survival rate differed across test plant species (F_7, 42_ = 5.10, *p* = 0.0003). Larval survival was highest on the native *L. polycarpa* and was statistically different from that on *L. palustris* and *C. amoena*, which supported intermediate and the lowest larval survival rates, respectively (Tukey’s HSD test, *p* ≤ 0.05). There was no difference in larval survival between the three exotic weeds. Mean larval development time did not differ between test plant species (F_7, 41_ = 1.77, *p* = 0.1206).

Oviposition and egg viability were monitored on the same eight test plant species that supported complete larval development (Table 1). However, *C. amoena* was omitted from the analyses because there was only one replicate: 379 eggs oviposited, with a 0.46 hatch rate. Total eggs per female differed between the remaining test plant species (F_6, 34_ = 9.06, *p* < 0.0001). The highest number of eggs was oviposited on the native *L. polycarpa*, which differed from *L. repens*, *L. palustris*, *L. peploides* subsp. *montevidensis*, and *L. hexapetala* (Tukey’s HSD test, *p* ≤ 0.05). The lowest number of eggs were oviposited on the native *L. repens*, which differed from *L. palustris*, *E. ciliatum* subsp. *ciliatum*, *L. peploides* subsp. *peploides*, and *L. hexapetala* (Tukey’s HSD test, *p* ≤ 0.05). Oviposition did not differ between the three exotic weeds, or between the exotic weeds and two native species (*L. palustris* and *E. ciliatum* subsp. *ciliatum*) (Tukey’s HSD test, *p* > 0.05). Correspondingly, egg hatching rate did not differ between test plant species (F_6, 34_ = 0.69, *p* = 0.6573).

### 3.3. Multiple-Choice Host Range Tests

*Lysathia flavipes* oviposited on all 11 plant species tested (Table 2), but the number of eggs oviposited per plant differed between species (F_10, 21.48_ = 6.70, *p* < 0.0001). This difference was attributed to *C. amoena*, which received the highest number of eggs and differed from *L. palustris*, *E. canum*, *L. decurrens*, and *L. peploides* subsp. *montevidensis* (Tukey’s HSD test, *p* ≤ 0.05). *L. decurrens* differed from *O. elata* subsp. *hookeri* (*p* = 0.03). Of the three exotic weeds, *L. flavipes* preferred to oviposit on *L. peploides* subsp. *peploides* over *L. peploides* subsp. *montevidensis* (*p* = 0.04), but there was no difference between *L. peploides* subsp. *montevidensis* and *L. hexapetala* in the number of eggs oviposited (*p* = 0.99). Adult feeding damage was observed on all test plant species, except *L. decurrens* and *E. canum*, and minimally on *O. elata* subsp. *hookeri*.

## 4. Discussion

The life-history characteristics and host range of *L. flavipes* were studied to determine if the flea beetle is a suitable biological control agent of invasive *Ludwigia* spp. in the USA. Particular interest was placed on testing *L. flavipes* to complete the work started by Cordo and DeLoach [20] and because of the success of other flea beetles in weed biological control programs, including *Euphorbia esula* L. (leafy spurge), *Alternanthera philoxeroides* (Mart.) Griseb. (alligatorweed), *Myriophyllum aquaticum* (Vell.) Verdc. (parrot feather), and *Jacobaea vulgaris* Gaertn. (tansy ragwort) [23,24,25,26]. Beyond biological control, however, these data also have relevance to general biological parameters of the South American herbivore. Development times of immature stages on *L. hexapetala* were similar those on *M. aquaticum*, with generation time from egg to adult approximately 20 d on both host plants [20]. The size of the *L. flavipes* females (ca. 8 mm) and eggs (ca. 0.7 mm) are longer than the conspecific *L. ludoviciana*, which are 4–5 mm and ca. 0.5 mm, respectively [27]. The generation time of *L. flavipes* also appears at least seven days faster than *L. ludoviciana*, although direct comparisons are complicated by different temperature conditions [27]. Possibly the greatest disparity between these two closely related species is their respective reproductive performance and longevity. The number of eggs oviposited by *L. flavipes* during a female’s lifetime averaged over 1500 (longevity: 90 d), as compared to that of *L. ludoviciana* which ranged from 5 to 142 (longevity: 57 d). This fast generation time and high fecundity greatly facilitated rearing and experimentation with *L. flavipes* during host-specificity testing.

We found no evidence to support the hypothesis that *L. flavipes* is host specific to species within the *Ludwigia* section *Jussiaea*, including the target weeds *L. hexapetala*, *L. peploides* subsp. *peploides*, and *L. peploides* subsp. *montevidensis*. Under no-choice conditions, *L. flavipes* larvae fed and completed development on the three target weed species but also successfully developed on five of the seven native plant species tested. Additionally, there was no difference in *L. flavipes* survival or development time when comparing these eight host species (Table 1). Interestingly, higher levels of variability in survivorship was observed between *Ludwigia* species as compared to other confamilials. It is noteworthy that *L. flavipes* was unable to develop on *L. decurrens* as compared to more distantly related hosts. 

While larval survival and development provides important insights into host specificity, comparing adult fitness between individuals reared on different species can reveal sublethal effects of suboptimal hosts. However, no-choice oviposition tests demonstrate that *L. flavipes* females readily oviposited viable eggs on the same plant species that supported their development. In terms of number of eggs oviposited, *L. flavipes* females did not distinguish between the three exotic weeds and two native plant species (*L. palustris* and *E. ciliatum* subsp. *ciliatum*). Surprisingly, the highest number of eggs were observed on the native *L. polycarpa*. Egg hatching rates did not differ between the test plant species, suggesting no apparent decrease in fitness over a generation of feeding exclusively on the test plant species. It is surmised from these data that several native plants included in this study are likely to support sustained *L. flavipes* populations for more than one generation. Collectively, these data also indicate that the physiological host range of *L. flavipes* does not mirror the phylogenetic relationship of the *Ludwigia* species and their more distant relatives [18]. 

While larvae may lack host specificity, as shown for *L. flavipes*, females can restrict host use through selective oviposition. Therefore, multiple-choice tests were conducted to provide insights into the herbivore’s ovipositional host plant selection preferences. Herein, however, *L. flavipes* females did not demonstrate a strong ovipositional preference for species in the *Jussiaea* over more distantly related species (Table 2). As with larval development and survival, female ovipositional patterns did not correlate with host phylogenetic relatedness, as evidenced by the highly variable results for species within the *Jussiaea* as well as between confamilials. When provided a choice, *L. flavipes* placed markedly more eggs on *C. amoena* as compared to *L. hexapetala*, the host from which the insects were originally collected in South America. Additionally, *L. flavipes* also oviposited on species that do not support development. The flea beetle oviposited on *L. decurrens*, *E. canum*, and *O. elata* subsp. *hookeri*, yet larval development tests showed that the larvae cannot complete development on these species. These data provide strong evidence that *L. flavipes* females oviposit broadly among hosts that range from optimal to unacceptable suitability for larval survival.

Collectively, these data indicate that *L. flavipes* is not a specialist of the *Jussiaea* or *Ludwigia* but is a more generalist feeding herbivore. The findings are consistent with field observations of *L. flavipes* by Vogt and Cordo [28], who recorded adults feeding on *Ludwigia peploides* (Kunth) P. H. Raven and *Myriophyllum brasiliense* Cambess (=*M. aquaticum*). Cordo and DeLoach [20] also documented heavy adult feeding by *L. flavipes* on leaf discs of *L. peploides* and *M. aquaticum*, as well as slight feeding on species from four other genera: *Salvinia auriculata* Aubl. (salvinia), *Spirodela intermedia* W.D.J. Koch (giant duckweed), *Beta vulgaris* L. (leaf beet), and *Brassica oleracea* L. (cabbage). *Lysathia flavipes* adults have also been recorded in association with other species in the Onagraceae in Argentina: *Oenothera indecora* Cambess, *Oenothera rosea* L’Hér. ex Aiton, and *Oenothera glazioviana* Micheli (=*O. erythrosepala* Borbás) [29]. Hernández and Cabrera Walsh [21] observed feeding by a *Lysathia* spp., which is likely to be *L. flavipes*, on *L. grandiflora*, *Ludwigia elegans* (Cambess.) H. Hara, *Ludwigia leptocarpa* (Nutt.) H. Hara, and *Ludwigia bonariensis* (Micheli) H. Hara in Argentina. The data reported herein, however, are the first to quantify larval developmental parameters and oviposition behaviors of the flea beetle, which are critical for estimating the herbivore’s host range.

Although our results demonstrate that *L. flavipes* is not a suitable biological control agent for invasive *Ludwigia* spp. in the USA, these should not be extended to presume *L. flavip*es is equally unsuitable for biological control in other parts of the world where exotic *Ludwigia* spp. are also problematic (e.g., European countries). Flea beetles have been among the most effective weed biological control agents worldwide, including another *Lysathia* spp. introduced into South Africa from Brazil in 1994 to control *M. aquaticum* [30]. *Lysathia flavipes* may still be considered for introduction elsewhere and these data can guide future host range testing as well as facilitate the rearing and handling of *Lysathia* spp. in general.

## Figures and Tables

**Table 1:** Mean ± 1SE (*n*) larval survival and development (1st instar to adult), oviposition, and egg viability of *Lysathia flavipes* on exotic *Ludwigia* and native test plant species in no-choice host range tests.

Test-Plant	Larval Survivorship (Prop.) ^4^	Larval Development (Days)	Number of Eggs Per Female	Egg Hatching Rate Per Female
*Ludwigia* *hexapetala* ^1^	0.63 ± 0.07 (15) abc	20.8 ± 0.9 (14) a	154.1 ± 22.2 (12) b	0.62 ± 0.08 (9) a
*L. peploides* subsp. *peploides* ^1^	0.85 ± 0.06 (5) ab	18.7 ± 1.2 (5) a	178.8 ± 5.8 (4) ab	0.65 ± 0.17 (4) a
*L. peploides* subsp. *montevidensis* ^1^	0.75 ± 0.08 (5) abc	17.5 ± 0.6 (5) a	109.8 ± 37.2 (5) bc	0.57 ± 0.16 (5) a
*L. decurrens* ^2^	0 (5)			
*L. polycarpa* ^3^	0.95 ± 0.05 (5) a	20.9 ± 0.9 (5) a	325.6 ± 44.4 (5) a	0.59 ± 0.06 (5) a
*L. repens* ^3^	0.85 ± 0.06 (5) ab	21.1 ± 0.3 (5) a	25 ± 4.1 (5) c	0.82 ± 0.04 (5) a
*L. palustris* ^3^	0.45 ± 0.09 (5) bc	21.6 ± 1.3 (5) a	128.5 ± 22.5 (4) b	0.68 ± 0.10 (5) a
*Epilobium ciliatum* subsp. *ciliatum* ^3^	0.85 ± 0.06 (5) ab	19.2 ± 0.3 (5) a	161.2 ± 27.5 (5) ab	0.43 ± 0.11 (5) a
*E. canum* ^3^	0 (5)			
*Clarkia amoena* ^3^	0.25 ± 0 (5) c	20 ± 1.2 (5) a		
*Oenothera elata* subsp. *hookeri* ^3^	0 (5)			

^1^ Target weed; ^2^ Exotic weed; ^3^ Native species. ^4^ Means in a column followed by different lowercase letters are significantly different (*p* ≤ 0.05; ANOVA and Tukey’s HSD test).

**Table 2:** Mean ± 1SE (*n*) eggs oviposited by *Lysathia flavipes* on exotic *Ludwigia* and native test plant species in multiple-choice host range tests.

Test-Plant	Number of Eggs Per Test Plant ^4^	Range
*Ludwigia* *hexapetala* ^1^	44.1 ± 8.8 (15) abcde	0–96
*L. peploides* subsp. *peploides* ^1^	72.4 ± 15.9 (5) abd	31–125
*L. peploides* subsp. *montevidensis* ^1^	6 ± 3.7 (5) ce	0–15
*L. decurrens* ^2^	3.8 ± 3.1 (5) de	0–16
*L. polycarpa* ^3^	28.4 ± 13 (5) abcde	0–66
*L. repens* ^3^	58 ± 14.4 (5) abcde	23–90
*L. palustris* ^3^	45.8 ± 18.3 (5) bcde	7–106
*Epilobium ciliatum* subsp. *ciliatum* ^3^	43 ± 17.0 (5) abcde	20–110
*E. canum* ^3^	2.4 ± 2.4 (5) bcde	0–12
*Clarkia amoena* ^3^	171.6 ± 53.3 (5) a	96–381
*Oenothera elata* subsp. *hookeri* ^3^	69.2 ± 15.0 (5) abc	44–124

^1^ Target weed; ^2^ Exotic weed; ^3^ Native species. ^4^ Means in a column followed by different lowercase letters are significantly different (*p* ≤ 0.05; General Linear Model and Tukey’s HSD test).

## Data Availability

The data presented in this study are available on request from the corresponding author.
